# Electrospun Polybenzimidazole Membranes: Fabrication and Fine-Tuning Through Physical and Statistical Approaches

**DOI:** 10.3390/polym17121594

**Published:** 2025-06-06

**Authors:** Emmanuel De Gregorio, Giuseppina Roviello, Valentina Naticchioni, Viviana Cigolotti, Alfonso Pozio, Luis Alexander Hein, Carlo De Luca, Claudio Ferone, Antonio Rinaldi, Oreste Tarallo

**Affiliations:** 1 Department of Engineering, University of Naples ‘Parthenope’, Centro Direzionale, Isola C4, 80143 Napoli, Italy; emmanuel.degregorio001@studenti.uniparthenope.it (E.D.G.); giuseppina.roviello@uniparthenope.it (G.R.); claudio.ferone@uniparthenope.it (C.F.); 2 Department of Energy Technologies and Renewable Sources (TERIN), ENEA, Via Anguillarese 301, 00123 Rome, Italy; valentina.naticchioni@enea.it (V.N.); viviana.cigolotti@enea.it (V.C.); alfonso.pozio@enea.it (A.P.); carlo.deluca2@unina.it (C.D.L.); 3 Nanofaber s.r.l., Via Anguillarese 301, 00123 Rome, Italy; luis.hein@nanofaber.com; 4 Department of Chemical Sciences, Università degli Studi di Napoli Federico II, Complesso Universitario di Monte Sant’Angelo, Via Cintia, 80126 Napoli, Italy

**Keywords:** polybenzimidazole membranes, electrospinning, Design of Experiment (DOE), analysis of variance (ANOVA)

## Abstract

Polybenzimidazole (PBI), a high-performance polymer known for its exceptional thermal stability and chemical resistance, was processed by solution electrospinning to manufacture fibrous non-woven membranes. The process was repeated under different conditions by adjusting four main settings: the polymer solution concentration, the flow rate, the voltage applied between the needle and the collector, and the separating distance. To clarify the interplay between process parameters and material properties, a Design of Experiment (DOE) approach was used to systematically analyze the effects of said parameters on microstructural properties, including fiber diameter, porosity, and air permeability, pointing out that the increase in viscosity improves fiber uniformity, while optimizing the applied voltage and the needle–collector distance enhances jet stability and solvent evaporation, crucial for defect-free fibrous microstructures. Post-processing via calendering further refined the membrane texture and properties, for example by reducing porosity and air permeability without significantly altering the fibrous morphology, particularly at low lamination ratios. Thermal and mechanical evaluations highlighted that the obtained electrospun PBI membranes exhibited enhanced flexibility, but lower tensile strength compared to cast films due to the underlying open pore microstructure. This integrated approach—combining experimental characterization, DOE-guided optimization, and post-processing via calendering—provides a systematic framework for tailoring PBI membranes for specific applications, such as filtration, fuel cells, and molecular sieving. The findings highlight the potential of PBI-based electrospun membranes as versatile materials, offering high thermal stability, chemical resistance, and tunable properties, thereby establishing a foundation for further innovation in advanced polymeric membrane design and applications for energy and sustainability.

## 1. Introduction

**Scheme 1:**
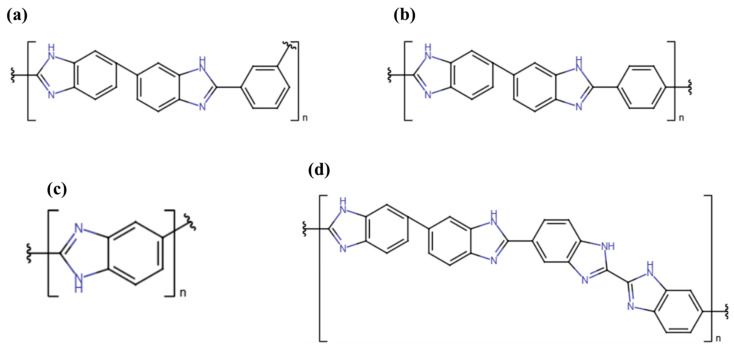
Chemical structure of different polybenzimidazoles: (**a**) poly(2,2′-*m*-phenylene−5,5′-bibenzimidazole), (**b**) poly(2,2′-(*p*-phenylene)5,5′-bibenzimidazole), (**c**) poly(2,5-benzimidazole), and (**d**) isomeric-Poly(2,5-benzimidazole).

Polybenzimidazoles represent a family of heterocyclic polymers characterized by a condensed aromatic structure and imidazole ring (Scheme 1), imparting them a basic character.

**Figure 1:**
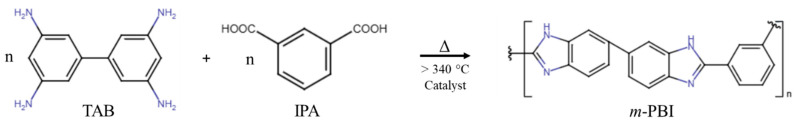
*m*-PBI synthesis route (TAB: tetraaminobiphenyl; IPA: isophthalic acid): the reaction is typically conducted in polyphosphoric acid (PPA), which acts both as a dehydrating agent and as a catalyst. The mixture is heated in stages—initially to approximately 200 °C to promote the formation of imidazole oligomers, and then to temperatures above 300 °C to complete cyclization and enable polymer chain growth.

Among these, the acronym PBI typically refers to the only commercially available polybenzimidazole polymer: poly [2,2′-(*m*-phenylene)−5,5′-bibenzimidazole] (also indicated as *m*-PBI, Figure 1). PBI is a polymer with remarkable thermal, chemical and mechanical properties. It has a T_g_ of 425 °C and a decomposition temperature > 600 °C [1]. The synthesis reaction for this material was developed in the 1960s [2] and consists of a condensation polymerization process between 3,3′-diaminobenzidine and terephthalic acid (Figure 1). Generally, the PBI is insoluble or poorly soluble in almost all major organic solvents but, depending on the nature of the starting dicarboxylic acid (DCAs), it exhibits good solubility in high-boiling polar aprotic solvents such as N,N-dimethylacetamide (DMAc), dimethyl sulfoxide (DMSO) and N-methyl−2-pyrrolidone (NMP) [3]. Furthermore, the addition of heteroatoms in the polymer backbone (principally oxygen and nitrogen) significantly increases the solubility of PBI even in low-boiling and polar solvents [4] such as formic acid (FA) [5].

Over the past decade, PBI has been widely studied and used due to its ability to interact with strong acids due to the presence of the nitrogen groups present in imidazole. For this reason, PBI membranes are generally ‘doped’, i.e., loaded with organic [6] and/or inorganic fillers [7] such as graphene oxide or silicon dioxide [8]. Due to its excellent chemical stability, mechanical strength, thermal stability, durability and low cost, PBI is used in the form of membranes for various purposes, especially at high temperatures and in aggressive environments. Furthermore, due to the close packing of the polymer chains deriving from the rigidity of the molecular structure and the strong hydrogen bonding between them, PBI membranes are characterized by very low gas permeability [9]. PBI can be used for different types of applications: (a) CO_2_ capture, due to its basic structure that promotes interactions with Lewis acids, even under anhydrous conditions [10]; (b) high-temperature fuel cells (HTPEMFCs), thanks to the fact that the impregnation of PBI with phosphoric acid (PA) makes the material ductile and highly conductive, even at temperatures above 100 °C and thus in the absence of water [11]; (c) electrodialysis, thanks to the promising transport rates for HCl, H_2_SO_4_ and HNO_3_ [12]; (d) osmosis, thanks to excellent chemical and mechanical stability even in harsh conditions [13].

Several techniques for manufacturing PBI membranes have been developed to date, such as blade casting and sol–gel [14]. Prominent among these is electrospinning, which is an advanced nanofiber production technique that can be used to produce polymeric membranes [15]. The main electrospinning advantage lies in the production of materials with a fibrous morphology, characterized by a high surface area-to-volume ratio and a reduced thickness (typically < 10 µm) [16]. In addition, physical parameters such as the porosity of the mat and average fiber diameter can be controlled depending on the characteristics of the capture or adsorption system and production requirements [17]. This morphology also results in improved mechanical properties of the material, as the fibrous structure with aligned and/or interconnected fibers provides high tensile strength and good flexibility, ensuring the integrity of the membrane during operation [18]. The formation of a fibrous matrix is strongly dependent on process parameters such as flow rate, ejector and collector voltages and needle–collector distance, but is also a function of the polymer–solvent pair and the environmental conditions of temperature and humidity [19].

As the electrospinning technique is rather complex and capable of generating a variety of porous structures, heuristic approaches based on statistical methods can be convenient and effective to optimize the microstructural parameters of the material such as porosity, gas permeability and the distribution of the mean diameter of the fiber affecting the impregnation capacity of the scaffold [20,21]. Statistical Design of Experiments (DOE) can be effectively used for this aim, to determine whether fine-tuning (input) process parameters allows the electrospinning process to produce (output) membranes that better suit a given application. On the other hand, the materials obtained by electrospinning can also be modified by post-processing physical treatments. One of these is calendering, representing a simple route for optimizing electrospun membranes by a thermo-mechanical treatment [22]. For example, this procedure allows fine-tuning of the morphological characteristics and porosity of membranes, crucial in sectors such as separation, filtration and adsorption.

This work aims to identify a robust set of process conditions for achieving PBI fibrous membranes using solution electrospinning and to illustrate the main effects and interactions of process parameters on the morphology of the resulting membrane. Furthermore, we discuss the possibility of fine-tuning the electrospinning process to produce PBI scaffolds for various engineering applications, ranging from filtration to batteries and/or fuel cell membranes.

## 2. Materials and Methods

### 2.1. Electrospinning of PBI and Design of Experiments

PBI solution (S26, PBI Product©, Charlotte, NC, USA, M_w_ = 28 kDa), with a viscosity of 2100 ± 200 Poise containing 26 %wt. of PBI in dimethylacetamide (DMAc, ≥99%, Sigma Aldrich, Burlington, MA, USA) and LiCl (4 %wt.), was used for the preparation of electrospun membranes. Several PBI solutions have been prepared at different concentrations by dilution in further DMAc. The electrospun fibers were manufactured with a pilot-scale electrospinning station (Fluidnatek LE100, Bioinicia SL, Valencia, Spain) equipped with a stationary planar collector (x-y plane) measuring 40 cm × 40 cm (approximately A6 sheet format) and with an environmental chamber capable of controlling temperature with an accuracy of 1 degree and humidity with a maximum error of 5%. Initial trials at 15 %wt. were run to find the environmental and process settings (Table 1) that yield a clear fibrous structure. Analysis showed that fiber shape depends strongly on temperature and humidity, and that stable flow appears only at high enough voltages and flow rates (see entry PBI_15_d, Table 1). Using these promising conditions as a starting point, we fixed temperature, humidity and collector voltage and set up a 2^k^ factorial design with k = 4. Spinning solutions at 14 and 16 %wt. were also prepared to refine the process. Each solution was mixed in a vortex for 20 min and then stirred for 24 h at room temperature to ensure complete homogeneity. During electrospinning, we adjusted the flow rate and spinning area so every sample had a uniform thickness.

For each process parameter—polymer solution concentration, ejector voltage, flow rate, and needle–collector distance—two levels were chosen, yielding 17 experiments in total (see Table 2).

Design of Experiment (DOE) is a systematic method to determine the relationship between factors affecting a process and the output of that process. Regression models play a crucial role in DOE analysis as they help quantify the effects of different factors and their interactions on the response variable. Linear regression models describe the dependence of each output variable (*Y*) on the process variables (*X*). The general form of the regression model is described by the following equation (Equation (1)) in the case of four regressors:

(1) $$Y=\beta_{0}+\beta_{i}X_{i}+\beta_{ij}X_{i}X_{j}+\beta_{ijk}X_{i}X_{j}X_{k}+\beta_{ijkm}X_{i}X_{j}X_{k}X_{m}\to \;i,j,k,m=1\mathrm{to}4$$

The primary objective of this approach is to identify the optimal descriptive model using a limited number of parameters and experiments, which can be directly and reproducibly applied to produce materials with desired chemical–physical properties, in accordance with the employment application. The electrospinning process is strongly influenced by the operating conditions used (*X_i_*), which in turn have a significant impact on the chemical–physical properties of the membranes obtained (*Y_i_*). As described above, a 16 %wt. PBI solution in DMAc was prepared and kept stirring for 24 h at room conditions to ensure complete and homogeneous dissolution. Each membrane was obtained from a specific combination of *X_i_* parameters, which were implemented to produce a combinatorial full-factorial 2^4^ approach from Design of Experiment (DOE) that aimed to examine the influence of 4 process parameters shown in Table 3 on the microstructural properties of the membranes described in Table 4.

The parameters shown in Table 2 are coded using ‘+1’, ‘−1’ and ‘0’ to identify the highest, lowest and intermediate level of each factor: the model employs coded variables “X_j_*”, derived from the natural variables through linear transformations, as described in the formula (Equation (2)) where “HIGH” and “LOW” correspond to the maximum and minimum ranges as indicated previously. To minimize the impact of uncontrolled ambient variables, the order of treatments in Table 2 was randomized during the experiment.

(2) $$X_{j}^{*}=\frac{x_{i}-\bar{x_{i}}}{{(x}_{HIGH}-x_{LOW})/2}\;\;(j\;\mathrm{from}\;1\;\mathrm{to}\;4)$$

By using coded variables, it is possible to standardize the range and scale for each parameter, which simplifies the comparison of their individual effects on the outcome [23]. This approach also supports the creation of an orthogonal design, ensuring that each factor can be estimated independently without interference from the others [22]. As a result, these coded variables play a critical role in the regression model by enabling the straightforward and immediate identification of significant terms. *p*-values are then used to assess whether the influence of each coded variable or their interactions is statistically significant. Specifically, if a coded variable yields a *p*-value below a set threshold (such as 0.05 or 0.10), it indicates that the factor has a meaningful impact on the response. Conversely, higher *p*-values imply that the factor’s effect might be negligible, warranting its removal from the model to streamline the analysis. By leveraging *p*-values to fine-tune the model, one can achieve a more parsimonious model that still effectively accounts for the variability in the response variable [21]. The ANOVA analysis for the subsequent models was carried out using JMP Pro software (version 17, SAS Institute, Cary, NC, USA) with a 10% significance level, thereby facilitating a clear distinction between significant and non-significant terms. To evaluate the quality of the experimental design derived from the ANOVA, the coefficient of determination (*R*^2^) is employed as the primary indicator of fit quality, reflecting the proportion of variance explained by the model. However, because *R*^2^ typically increases with the addition of more parameters, it is also essential to consider the adjusted *R*^2^ (*R*^2^*_adj_*) (see Equation (3)).

(3) $$R_{adj}^{2}=1-(1-R^{2})\frac{p}{n-p-1}$$

The value is consistently lower than *R*^2^ and is determined by the ratio of the number of predictors (*p*) to the number of data points (*n*) [21].

### 2.2. Sample Characterization

#### 2.2.1. Scanning Electron Microscopy (SEM) Evaluation: Morphological Characterization and Fiber Analysis

The morphology of the electrospun samples was examined using the Scanning Electron Microscopy technique. SEM analysis was carried out using a Nova Nanosem 450 (FEI, Austin, TX, USA) on the pristine PBI-based scaffolds, metallized with Pd/Au. The acceleration potential used for image acquisition is in the 2–5 kV range. The fiber diameter and the distribution of the beads were measured with ImageJ software (version 1.54, Rasband, W.S., Image J, U.S. Nation Institute of Health, Bethesda, MD, USA). The fiber diameter mean values (MFD) and root mean square errors (RMS) distributions were obtained by analyzing at least 50 random spots over at least three different micrographs for each sample (5000× magnification).

#### 2.2.2. Porosity and Air Permeability

The porosity of the electrospun materials was determined gravimetrically on membranes with dimensions 2 × 2 cm^2^ using the liquid displacement technique [24] in ethanol (Sigma Aldrich, 99% anhydrous), which was chosen because it permeates through the membrane without generating interactions or swelling. The percentage porosity (ε%) was calculated from the following equation (Equation (4)):

(4) $$\varepsilon \%=\frac{\left(m_{3}-m_{4}-m_{1}\right)}{\left(m_{2}-m_{4}\right)}\;\cdot 100$$

where *m*_1_ refers to the mass of the dry membrane as such, *m*_2_ refers to the mass of a graduated flask containing ethanol, *m*_3_ is the mass of the same system containing the PBI membrane to which the excess ethanol due to the displaced excess volume caused by the presence of the membranes has been carefully removed, m_4_ is the mass of the graduated system without the membrane. The determination of air permeability for PBI-based electrospun membranes was conducted using a Gurley densimeter (Model 4320, Troy, NY, USA). All electrospun samples were cut into 2 × 2 cm^2^ to ensure the passage of a volume (*V*) of air equal to 100 cm^3^ through the required active area (*A*) of 1.58 cm^2^ at a time (*t*). Permeability (*P*) was calculated for each sample from Equation (5) and is expressed in cm/s:

(5) $$P=\frac{V}{A\;\cdot \;t}$$

#### 2.2.3. X-Ray Diffraction

The study of the PBI electrospun membrane structure was carried out through a Wide-Angle X-ray Diffraction (WAXD) technique using the Malvern Panalytical (Malvern, Worcestershire, UK) Empyrean multipurpose diffractometer using Cu Kα (λ = 1.5418 Å) by using a continuous scan of the diffraction angle 2θ from 5–40° with a scan rate of 0.05°/s.

#### 2.2.4. Attenuated Total Reflectance Fourier-Transform Infrared Spectroscopy (ATR-FTIR)

The pristine electrospun PBI-based membrane was characterized through ATR-FTIR Spectroscopy. This analysis was performed using an Anton Paar (Rivoli, Italy) Lyza 7000. The scans were recorded using a diamond crystal cell through 36 scans at a resolution of 4 cm^−1^. The sample was cut into 1 × 1 cm^2^ by applying the required torque to keep in contact the membrane with the crystal.

#### 2.2.5. Thermogravimetric Analysis (TGA)

The thermal degradation of PBI-based electrospun membranes was evaluated using the TA Instrument (Sesto San Giovanni, Italy)—SDT650. Approximately 8–10 mg of sample were placed in an alumina crucible and tested under a N_2_ atmosphere (20 mL/min) with a heating rate of 10 °C/min up to a temperature of 800 °C.

#### 2.2.6. Mechanical Tests

Mechanical properties were performed at room temperature by using the Instron (Norwood, MA, USA) 5566H1543 machine to determine the tensile strength, Young’s modulus and the elongation at break according to standard ASTM D638 [25]. The dimensions of the testing samples were 100 mm × 10 mm, and the thickness of the samples was 100 µm. The deformation speed was adjusted to 10 times the initial gauge length (L_0_) per minute, resulting in a strain rate of v = 10 min^−1^ for measuring the stress–strain curves and subsequently determining the tensile parameters up to failure. For the assessment of Young’s moduli, the speed was set to 0.1 × L_0_ per minute, corresponding to v = 0.1 min^−1^.

### 2.3. Calendering

The calendering process involves the use of heated rollers to compress and flatten the membranes, enhancing their mechanical properties and surface characteristics. The thickness of the as-spun polybenzimidazole membrane was first mapped on 50 different spots to identify an initial average thickness (IT) with a digital micrometer. Then, the material was placed between two (near incompressible compared to the electrospun membrane) Mylar supports and subjected to calendering using a Hot Roller Press Machine (model 200L, by TOB Machine, Xiamen City, Fujian Province, China). The distance between the two pressing rollers was set as a function of the initial thickness of the electrospun membrane by defining a “lamination ratio” (LR), which represents the ratio between the initial membrane thickness and the nominal target value (FT). LR = 2 and 3 were applied to the as-spun membranes in order to verify the effect of the treatment on air permeation rate and porosity.

## 3. Results

### 3.1. Electrospinning Process: Morphological Study

**Figure 2:**
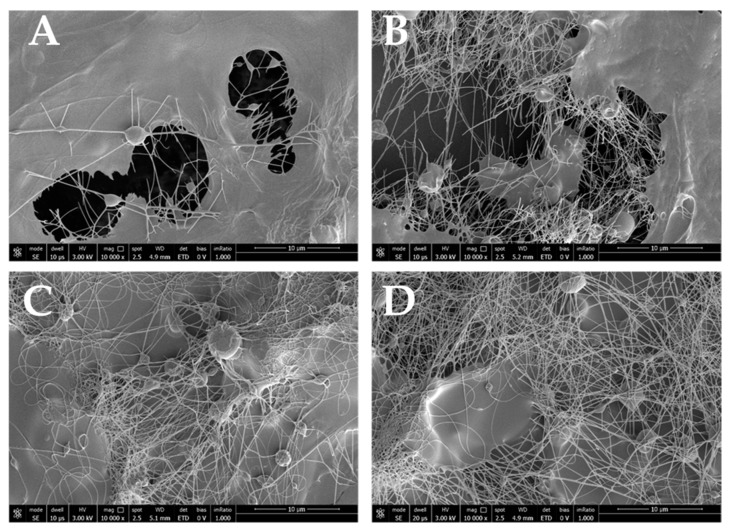
SEM micrographs at 10,000× magnification of preliminary sample mats produced using a 15 %wt. PBI solution in DMAc. Sample obtained at 25 °C and 40% RH (**A**), at 35 °C and 25% RH (**B**), at 40 °C and 25% RH (**C**), and at 45 °C and 20% RH (**D**).

The variation in electrospinning parameters is crucial due to their precise correlation with the chemical, physical, and morphological properties of the electrospun membrane. Specifically, the influence of different electrospinning parameters on the morphology of the samples was investigated by analyzing their surfaces using SEM microscopy. The micrographs shown in Figure 2 refer to preliminary electrospinning tests conducted on a 15 %wt. PBI solution in DMAc. Moving from micrograph A to D, the effect of temperature and humidity in the electrospinning chamber on the final material morphology becomes evident, with the formation of interconnected fibers interspersed with dense regions. In particular, under standard room conditions (Figure 2, sample A), the whipping motion required to form fibers is not achieved. Instead, the sample exhibits a dense morphology interconnected with small fibrous spots and defects (beads) across its surface. This is likely due to the high flow rate, which reduces the charge density at the ejector, preventing the formation of a continuous polymer jet. It is well known that increasing the feed rate decreases the charge density, while higher charge density induces jet bending instabilities, leading to the formation of thinner fibers [26]. Additionally, a high feed rate hinders the complete evaporation of solvent molecules, resulting in the presence of beads. However, by modifying the temperature, and relative humidity, and reducing the flow rate (as detailed in Table 1), the development of a fibrous structure becomes more pronounced, as seen in micrograph B of Figure 2. Adjustments to temperature and humidity significantly improved the sample morphology, producing well-interconnected fibrous regions deposited on a dense matrix. Despite these improvements, the samples still display bead-like spots, as observed in conditions C and D (Figure 2C and Figure 2D, respectively).

As expected, the SEM micrographs from the preliminary sample tests demonstrate that variations in electrospinning parameters are crucial in the formation of the fibrous structure. Consequently, electrospinning production run were conducted using a Design of Experiments (DOE) approach to systematically and efficiently plan experiments, enabling the correlation of experimental outcomes with specific electrospinning parameters. These parameters, selected from a range tested at a 15 %wt. polymer solution concentration, included ejector voltage, needle–collector distance, and flow rate, as outlined in Table 1. Also in these cases, SEM micrographs were analyzed to evaluate the impact of each individual process parameter, aiming to establish a morphological correlation across the samples. In the following paragraphs, the morphology of selected samples obtained by this approach have been discussed.

#### 3.1.1. Effects of Voltage and Collector Distance

The effects of voltage and collector distance were studied by the 14 %wt. samples characterization. Figure S1 shows SEM micrographs of the 14 %wt. samples obtained at a flow rate of 1 mL/h but at different ejector voltages and different needle–collector distances (see Table 1 for experimental conditions). The pictures show a different situation: samples PBI_E01 and PBI_E05 were obtained at the same voltage (V = 17 kV) but at different needle–collector distances (135 and 115 mm, respectively). The micrographs suggest that the decrease in distance, at the same voltage and flow rate, does not guarantee the continuity of the polymer jet likely due to a repulsion phenomenon between the charged particles, as shown by the sample PBI_E05. In fact, on the other hand, by increasing the distance (e.g., PBI_E01), the sample structure appears quite different, with a well-defined and rather uniform morphology, which is much better than the PBI_E05 sample. This comparison highlights the high influence of the distance parameter in the electrospinning process of PBI that can modify all the properties of the resulting material.

In addition, also voltages and the flow rate show a high impact on the sample quality. For example, the excessive voltage (20 kV) in the PBI_E03 resulted in a sample with a not well-defined fibrous morphology that is full of defects. While high voltage promotes rapid solvent evaporation, the short distance might not provide sufficient time for complete evaporation. The combination of high voltage and low needle–collector distance did not allow the jet to solidify properly, resulting in a dense and non-uniform structure, as is evident in PBI_E07. In this last situation, it is clear that the polymer solution had no time to allow the solvent to evaporate completely, leading to wet fibers that during their solidification process, generated a densified microstructure, not fully fibrous. This supports the earlier statement that high applied voltages worsen the overall sample quality by degrading microstructural morphology and causing fiber coalescence.

Figure S2 shows the micrographs of the 14 %wt. electrospun samples that were obtained at a constant flow rate of 2 mL/h but at different voltages and needle–collector distances. The increase in flow rate makes it possible to obtain materials characterized by an interconnected fibrous morphology with the presence of beads and small dense areas, randomly distributed. Only the PBI_E08 sample shows a different behavior: working at a shorter distance (115 mm) from the collector, it is more affected by the ejector voltage. The discovery of dense structures that were not electrospun correctly confirmed the previous claim. In particular, the electric field strength between the needle and the collector is greater with a shorter distance, which can lead to instability in the jet. Moreover, the shorter distance can lead to needle clogging, especially if the polymer solution is not properly atomized and stretched. The result is the formation of unspun in-desiderate dense structures, as described for the previous samples with the same structured materials.

#### 3.1.2. Effects of Viscosity and Interactions with the Other Parameters

The increase in concentration (16 %wt.) resulted in a remarkable improvement in the final morphology of the samples. In particular, the polymeric solution has a higher viscosity than the materials synthesized and shown before (14 %wt.). It is already known that viscosity is a critical parameter in electrospinning because it can reduce the occurrence of bead defects in the fibers, producing smoother and more uniform samples [27]. In addition, higher viscosity helps stabilize the jet, reducing the chances of jet breakup and resulting in more consistent fiber diameters [28]. In particular, micrographs of the samples obtained at a constant flow rate of 1 mL/h can be seen in Figure S3. It is worth pointing out that both the voltages used, and the needle–collector distances allow the obtainment of homogeneous materials with good fibrous distribution. Sample PBI_E015, therefore, shows a large fibrous spot probably due to a sudden interruption of the flow during the electrospinning process that leads to a beads-like structure over the surface.

**Figure 3:**
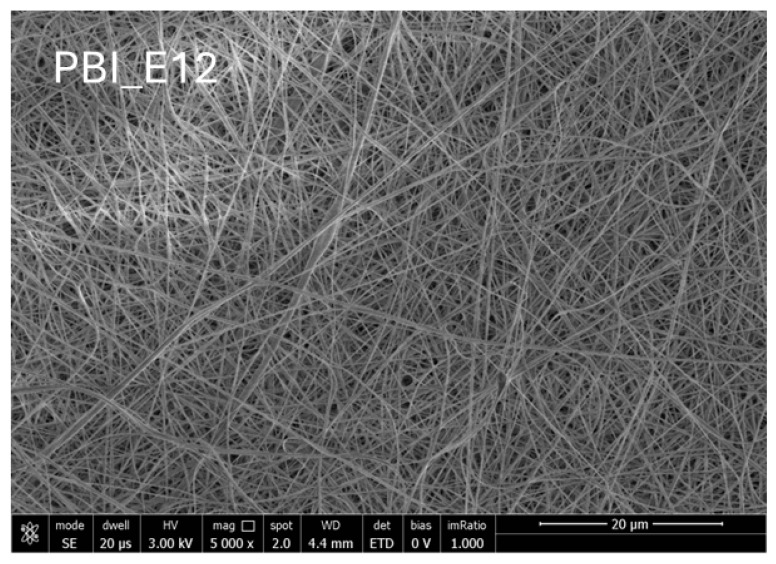
SEM micrograph of PBI_E12, produced from a 16 %wt. PBI solution using a flow rate of 2 mL/h, 20 kV at the ejector, and a needle–collector distance of 135 mm. This sample was chosen as the best from the experimental campaign and was used for all subsequent chemical, physical, and mechanical characterizations.

By increasing the flow rate (2 mL/h) it is possible to obtain materials with different morphology (Figure S4) and, at the same time, to understand the influence of the various process parameters. For example, the PBI_E10 sample shows a surface characterized by large, non-fibrous spots randomly interconnected by non-continuous fibers of irregular thickness. This can likely be attributed to the lower applied voltage, which allows for a larger needle–collector distance. The combination of low voltage and high distance results in significantly reduced electric field strength, which may cause difficulties in initiating and sustaining a stable jet stream. In support of this thesis, the PBI_E12 sample obtained at the same needle–collector distance but at a higher applied voltage shows a uniform and completely fibrous morphology, without any beads or narrow fibers defects as reported in Figure 3. The decrease in distance (115 mm) slightly affects the subsequent samples PBI_E014 and PBI_E016, which show a good fibrous surface but the presence of random beads and dense spots due to an incorrect ejection of the polymer solution.

The last sample analyzed in Figure S5, PBI_E17, was obtained under average electrospinning conditions in respect to the values used for all other samples, working with a 15 %wt. solution. The material is characterized by the presence of fibers spaced with dense dots greater than 1 µm in diameter that cover the entire surface of the sample. The lower viscosity of the sample and the use of a relatively high flow rate do not allow the formation of a continuous and stable jet, able to guarantee the correct evaporation of the solvent during the time of flight and, therefore, the formation of the fibers. The DOE model can be improved by incorporating intermediate tests, which is an effective strategy to improve the quality and reliability of experimental results. It helps not only to validate and optimize the model but also to ensure that the conclusions drawn are robust and well founded.

### 3.2. Fiber Diameter Distribution, Porosity and Air Permeability

**Figure 4:**
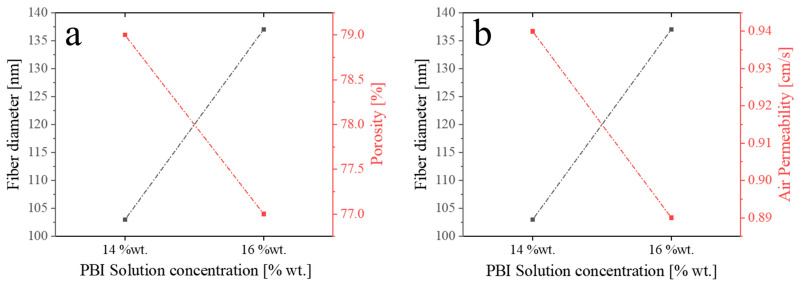
Variation in porosity (**a**) and air permeability (**b**) with the concentration of the PBI solution. Reported data points are obtained as the average of multiple measurements performed on each of the samples studied (see Table 5). Dashed lines are a guide for the eye.

In electrospun membranes, the relationships between fiber diameter, porosity and air permeability are interdependent [27]. These properties are crucial for the performance of the membrane in various applications such as filtration, sensors, and tissue engineering. The increase in fiber diameter leads to a reduction in the space between the fibers, in turn leading to a decrease in the overall porosity and permeation properties of the membrane [16,27]. The average values of these parameters are reported in Table 5. Figure 4 shows how the increase in concentration of the PBI solution (from 14 %wt. to 16 %wt.) leads to a decrease in porosity (Figure 4a) and air permeability (Figure 4b) with an overall increase in fiber diameter. The increase in fiber diameter is accompanied by a decrease in porosity that indicates a balance between the viscoelastic forces of the polymer and the physical structure of the fiber; at higher concentrations, the solution becomes more viscous, which may affect the polymer’s ability to “stretch” or “elongate” during the fiber formation process [29,30]. As expected, increasing the polymer concentration results in a higher viscosity of the electrospinning solution. This reduces the stretching of the jet under the electric field, producing fibers with larger diameters. Since the mass flow rate remains constant, fewer but thicker fibers are deposited. As a result, the total void volume (i.e., porosity) stays nearly unchanged: although the pores become slightly larger, they are fewer in number. This compensating effect maintains a similar overall porosity and air permeability, despite differences in fiber diameter and morphology.

### 3.3. Structural Characterization: WAXD, TGA and ATR-FTIR

**Figure 5:**
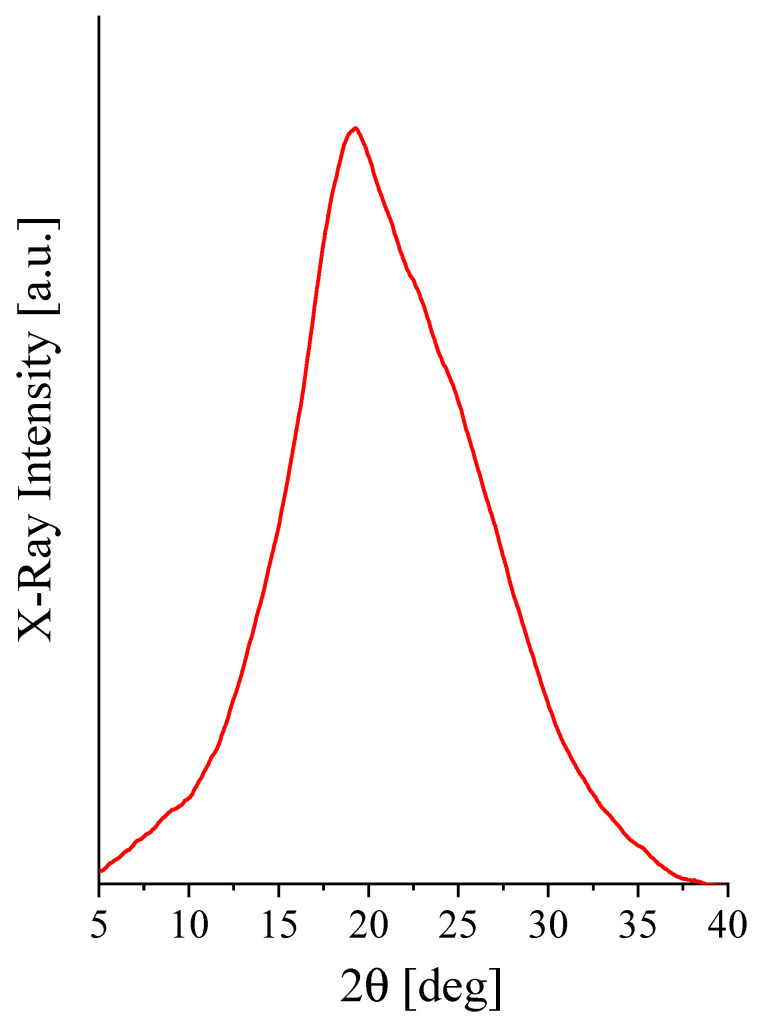
XRD pattern of PBI-based electrospun membrane. The pattern typically shows a broad diffraction peak, indicating a predominantly amorphous structure. This is consistent with the nature of electrospun polymer mats, where rapid solvent evaporation and fiber formation lead to less organized molecular packing. The presence of a broad peaks approximately 20–25° 2θ can be attributed to short-range ordering of the polymer chains [33].

The PBI electrospun membrane exhibits, as expected, an essentially amorphous structure, as shown by the WAXD diffraction spectrum in Figure 5. In particular, the sample shows a diffuse amorphous halo centered approximately 2θ~20° with the absence of any sharp peaks, as occurs in the polybenzimidazole film obtained by solution casting [31,32].

**Figure 6:**
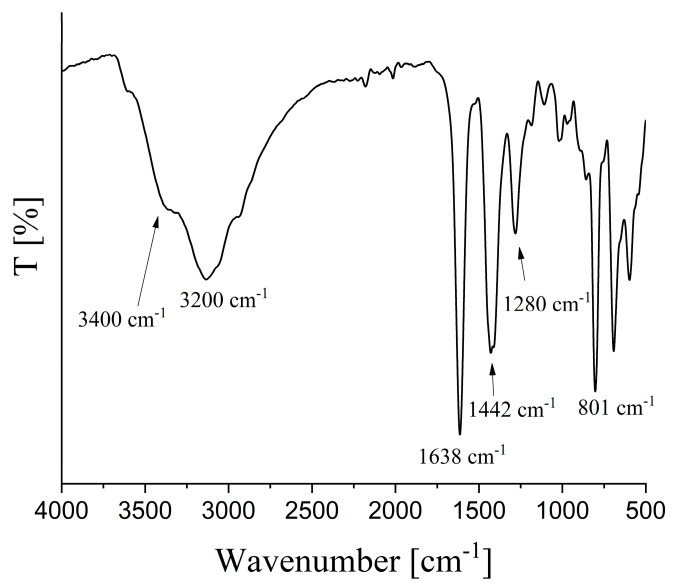
FTIR analysis of PBI electrospun membrane. Characteristic absorption bands are observed at approximately 3400 cm⁻^1^, corresponding to N-H stretching vibrations, indicating the presence of imidazole groups. Peaks near 1640 cm⁻^1^ and 1440 cm⁻^1^ are assigned to C=N and C=C stretching vibrations within the benzimidazole rings. The absence of significant peaks related to solvent residues (approximately 1650–1690 cm⁻^1^ and corresponding to carbonyl (C=O) stretching vibration) confirms effective removal of solvents during electrospinning.

FTIR spectra performed in ATR (Figure 6) display characteristic peaks related to aromatic C-H bending out-of-plane vibration (801 cm^−1^) and stretching vibrations due to imidazole’s C-N bond (1280 cm^−1^). The peaks in the range 1442–1638 cm^−1^ refer to the stretching of the C=N and C=C bonds while the signal at approximately 3200 cm^−1^ is attributable to the OH bond due to moisture as the material is slightly hydrophilic. The N-H functional group related to imidazole is indicated in the peak at 3400 cm^−1^.

**Figure 7:**
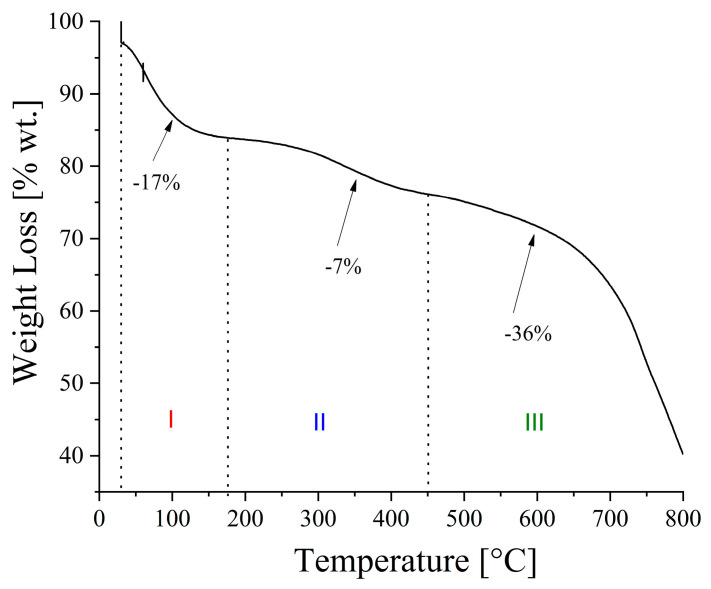
Thermogravimetric analysis of PBI-based electrospun membrane. A three-stage decomposition is apparent: region I, with a 17% weight loss, corresponds to moisture removal; region II, with an additional 7% weight loss, is due to minor residual solvents and degradation of N-H and N=C functional groups; region III, with a 40% weight loss, reflects the breakdown of the polymer backbone.

The thermogram in Figure 7 shows a 3-stage decomposition, typical for polybenzimidazoles [34]. The first weight loss (region I) of about 17 %wt. is associated with moisture and minor solvent residue, up to a temperature of about 130 °C. In the 150–450 °C range (region II), there is a further overall loss of 7 %wt. due to further solvent residues and deterioration of the N-H and N=C functional groups of the imidazole group of PBI. Finally, in the third stage of decomposition (region III), the polymer backbone is degraded up to a temperature of 800 °C with a residual of 40%.

### 3.4. Mechanical Test

**Figure 8:**
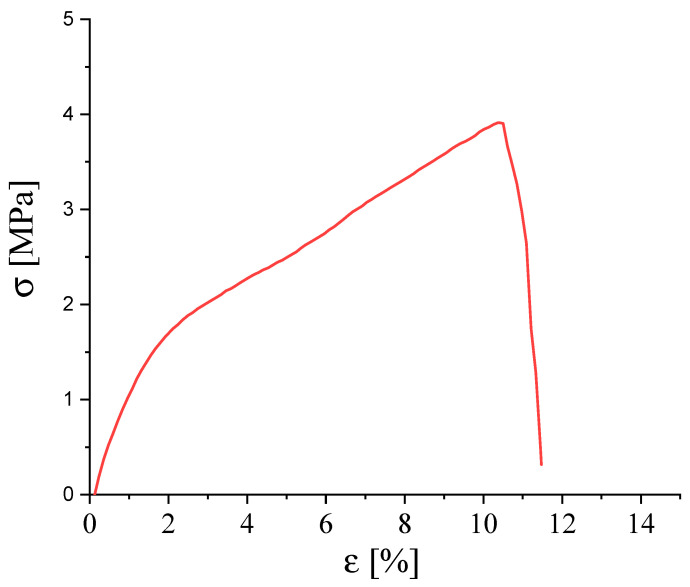
Stress–strain curves of PBI_E12 sample: the red curve is representative of an average value of the mechanical parameters.

From a mechanical point of view, the material shows a specific stress–strain curve (Figure 8). The PBI produced by the electrospun process exhibits unique mechanical properties due to the nanoscale fibrous structure. The curves describe a sufficiently ductile material with an initial linear stretch in which the membrane behaves elastically. The slope of this region is defined by Young’s modulus which has a value of 100 ± 20 MPa that is quite low compared to the PBI casting membrane (Table 6). Likewise, the tensile strength is 4 ± 0.3 MPa which is a low value that indicates that this particular morphology strongly influences the mechanical behavior of PBI membranes leading to a decrease in stiffness. The elongation at break is also lower, 12% ± 1, and this is surely due to the porous nature of the sample. It is well known that porous materials generally exhibit lower strength compared to their non-porous counterparts [31,35]. This is a result of pores acting as stress concentrators, resulting in localized high-stress areas that can lead to cracks and failure at lower loads. In addition, the presence of pores reduces the effective cross-sectional area that can carry a load, making the material less ductile. It is worth pointing out that the mechanical properties of PBI electrospun membranes are strongly dependent on their intrinsic morphology: fiber diameter and their spatial arrangement are fundamental parameters for the material mechanical improvement as they allow the applied load to be distributed along the alignment direction, improving load transfer between the fibers.

### 3.5. PBI-Based Electrospun Fine-Tuning

As previously mentioned, PBI is employed in a wide range of applications, from producing aerospace suits to capturing pollutants [37,38]. In this context, structural and morphological parameters such as material thickness and porosity play a crucial role, as they must be strategically tuned according to the intended application. To this end, we propose two complementary methodologies in the following sections. The first methodology involves the optimization of electrospinning parameters through a statistical approach based on ANOVA analysis. The second methodology focuses on the post-processing treatment of the electrospun membrane via calendering, primarily aimed to controlling porosity, gas permeability, and membrane thickness while preserving the characteristic fibrous structure of electrospun materials. The integration of these two approaches allows, in principle, the development of materials capable of meeting specific technical requirements, both in terms of mechanical strength and transport properties of gases and liquids.

#### 3.5.1. ANOVA Model for Y_i_

As previously reported, each membrane was obtained through a unique combination of *X*_i_ factors characterized by different values, identified as ‘levels’ that are coded using ‘+1’, ‘−1’ and ‘0’ to identify the highest, lowest and intermediate level of each factor (Table 7). To each interaction corresponds an experimental output *Y*_i_, directly correlated to the *X*_i_ factors employing an analysis of variance (ANOVA).

The best-proposed models for *Y*_1_, *Y*_2_, *Y*_3_ and *Y*_4_ are shown in Table 8 with the respective *R*^2^ and *R*^2^*_adj_* values. As shown, the predicted values have a good correlation R^2^ coefficient, also because the proposed models consider binary (e.g., *X*_1_*X*_2_) and ternary (e.g., *X*_1_*X*_2_*X*_3_) interactions that render a deep improvement in the final model. Details on the statistic models, the calculated residuals and the influence of each interaction on *Y_i_* responses can be found in the supporting information.

**Figure 9:**
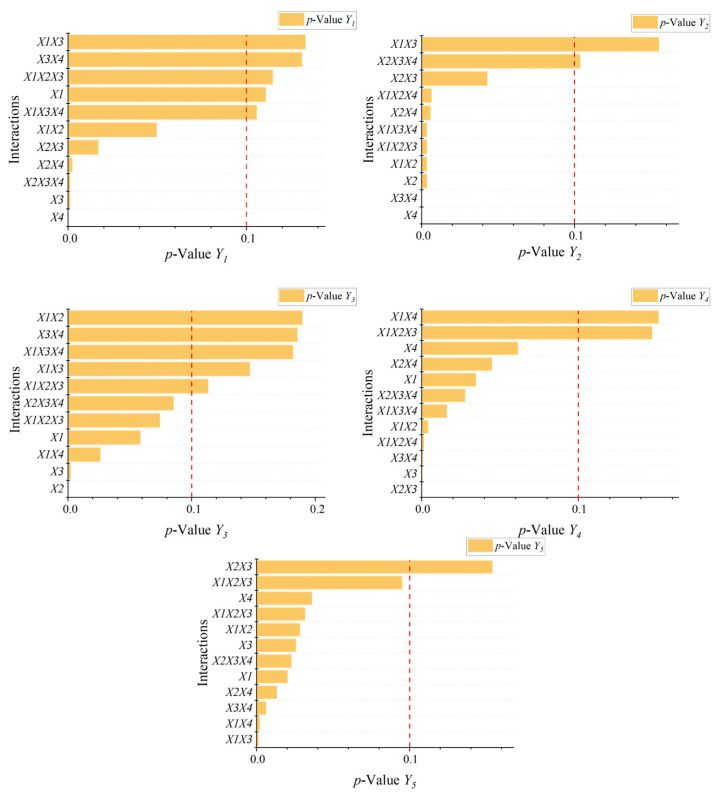
Bar diagram describing the *p*-values of each interaction for the studied outputs. The bars below the red lines are significant for the regression.

A *p*-value analysis was conducted to assess the effect of the different factors and their interactions on four response variables (*Y*_1_, *Y*_2_, *Y*_3_, *Y*_4_, *Y*_5_). The objective is to identify which factors have a statistically significant effect (*p*-value < 0.1) on each response variable and to construct regression models to describe these effects. Figure 9 shows the *p*-values of the different factors for the *Y_i_* response variables, with a red line indicating the significance level set at 0.1. Factors whose bars are below the red line are considered statistically significant. Relative to the *Y*_1_ and *Y*_2_ responses, significant facts include the variables *X*_2_, *X*_3_ and *X*_4_ and specific ternary interactions suggesting a combined influence on the output. The *R*^2^ parameter is sufficiently high (see Table 3) and appropriately describes the microstructural behavior of the material. The *p*-value analysis suggests a strong significance of parameters such as the needle–collector distance (*X*_3_), which influences the evaporation of the solvent and, thus, the formation of a continuous and regular fiber, and the concentration of the polymer solution (*X*_4_), which clearly defines the viscosity f the solution and the thickness of the fiber itself.

Ejector voltage (*X*_2_) and needle–collector distance (*X*_3_) are highly significant for air permeability (*Y*_3_), suggesting a significant effect on this response variable. This is a clear consequence of the electrospinning process, as the formation and distribution of fibers on the collector are dependent on the electrostatic action applied to the polymer solution, the needle tip and the velocity of the solvent evaporation, as described above.

Material porosity (*Y*_4_) shows the greatest number of significant factors, including both single effects and multiple interactions, indicating that the variability of *Y*_4_ is influenced by a combination of factors. It is important to emphasize that porosity, as well as the formation of the entirely fibrous material itself, is crucial for the use of polybenzimidazole-based membranes in various applications. Finally, the distribution of beads (*Y*_5_) is influenced by the flow rate and needle–collector distance, which affect the evaporation and distribution of fibers across the sample surface and, thus, the formation of microstructural defects (beads) and dense areas. This statistical study intends to provide a rational approach to the production of electrospun materials by appropriately setting the electrospinning parameters to obtain functional and reproducible materials with controlled physical and mechanical characteristics.

#### 3.5.2. Calendering Process

**Figure 10:**
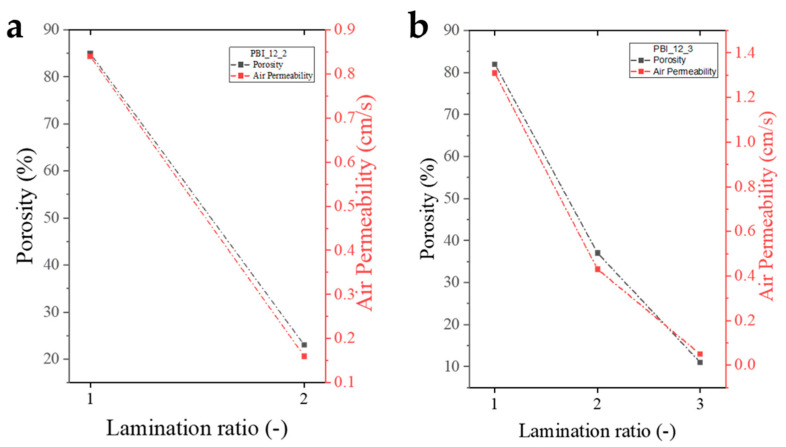
Effect of calendering process on air permeability and porosity of PBI-based electrospun membranes ((**a**), R = 2; (**b**), R = 3). Lines are only a guide for the eye.

Calendering resulted in extremely thin and homogeneous membranes (<25 µm) with significantly different permeation and porosity properties than the initial one. In particular, by increasing the rolling ratio and, thus, decreasing the distance between the rollers, the pressing process becomes extremely useful as it allows a reduction in air permeation rate and porosity by approximately 90% compared to the initial values (Figure 10). This is extremely relevant as the present work aims to produce and develop electrospun PBI membranes for various engineering applications through a special tuning of the morphological properties of the materials. Indeed, the reduction in porosity allows these materials to be used as true molecular sieves for gaseous effluents or enables the use of such membranes in electrochemical applications such as electrodialysis or fuel cells, processes in which low porosity prevents gaseous and/or liquid crossover phenomena.

**Figure 11:**
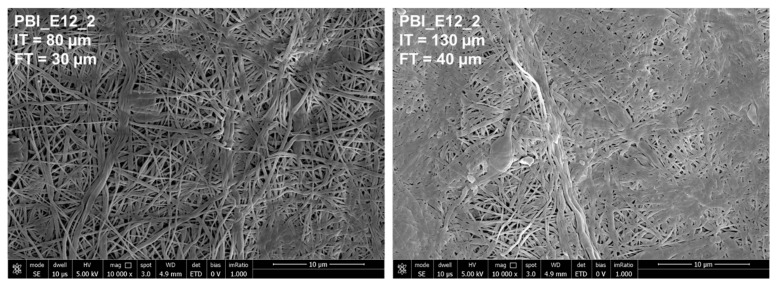
SEM micrographs of PBI-based electrospun membrane after calendering treatment using R = 2 (**left**) and R = 3 (**right**); IT = initial thickness; FT = final thickness.

It is important to point out that the calendering process does not significantly alter the fibrous morphology of the material, except for high lamination ratios: SEM analysis reveals the presence of distinct fibers on the material (Figure 11), which allows the high surface area and high adsorption capacity of the membrane to be exploited, a crucial point for the various applications. However, the increase in the lamination ratio makes it possible to observe spots in which a coalescence phenomenon is visible due to the mechanical action of pressing, which modifies the fibrous structure by altering its morphology, making the membrane, at least, close to one obtained, for example, by solvent casting. The analysis reported in this work has allowed the identification of an optimal lamination ratio that succeeds in guaranteeing the fibrous structure while maintaining the morphology as shown in Figure 11. It is worth highlighting that calendering is not only an effective post-processing technique for tuning the properties of electrospun PBI membranes but also serves as a valuable tool for further refining the physical and structural parameters of materials obtained through an ANOVA-based optimization of electrospinning conditions (see Section 2.1). This dual role enhances the ability to precisely tailor microstructural properties to meet specific application requirements.

## 4. Conclusions

The present work offers a comprehensive analysis of the electrospinning process for the production of polybenzimidazole-based membranes, highlighting the interplay between process parameters and the resulting material properties. This study demonstrates that the morphology, porosity, and functional performance of electrospun PBI membranes are significantly influenced by key factors such as polymer solution concentration, flow rate, ejector voltage, and needle–collector distance. Employing a Design of Experiment methodology, the relationships between these parameters were quantitatively established, enabling the development of statistically robust models to describe critical output variables, including fiber diameter, air permeability, porosity and surface defects. The results confirmed the importance of optimizing electrospinning conditions to achieve homogeneous fibrous structures. For instance, increasing the polymer solution concentration improved viscosity, which reduced the presence of bead defects and resulted in more uniform fibers. Similarly, precise control over the ejector voltage and needle–collector distance was found to be essential for maintaining jet stability and ensuring complete solvent evaporation, thereby facilitating the formation of continuous and defect-free fibers. Additionally, an ANOVA-based statistical approach and post-production methods, such as calendering, were suggested to fine-tune the morphological and microstructural properties of the electrospun membranes. In particular, it has been shown that, while preserving a fibrous structure, the calendering process effectively reduces porosity and air permeability, yielding thin, homogeneous membranes suitable for specialized applications such as energy storage or environmental remediation. To the best of our knowledge, this study is one of a handful of reports dealing with the production of PBI-based membranes made by electrospinning. Through a rational approach, the combination of statistical methods of Design of Experiments and calendering post-processing proved that it is possible to achieve a controlled modulation of processing parameters to fine-tune the physicomechanical properties of the resulting membrane material. This strategy enables the development of membranes with optimized characteristics, making them suitable for a wide range of high-performance applications.

## Figures and Tables

**Table 1:** Electrospinning parameters for PBI trial samples: temperature, relative humidity, ejector and collector voltages, needle–collector distance, and solution flow rate.

Sample	Temperature [°C]	Relative Humidity [%]	V Ejector [kV]	V Collector [kV]	Needle–Collector Distance [mm]	Flow Rate [mL/h]
PBI_15_A	25	40	20	−20	125	5
PBI_15_B	35	25	20	−20	125	0.4
PBI_15_C	40	25	20	−20	125	1
PBI_15_D	45	20	20	−20	125	1

**Table 2:** Electrospinning conditions obtained by perturbing the initial trials set according to a 2^4^ factorial scheme.

Sample	Flow Rate [mL/h]	V Ejector [kV]	Height [mm]	Concentration [%wt.]	Temperature [°C]	Humidity [%]	V Collector [kV]
PBI_E01	1	17	135	14	45	20	−20
PBI_E02	2	17	135	14	45	20	−20
PBI_E03	1	20	135	14	45	20	−20
PBI_E04	2	20	135	14	45	20	−20
PBI_E05	1	17	115	14	45	20	−20
PBI_E06	2	17	115	14	45	20	−20
PBI_E07	1	20	115	14	45	20	−20
PBI_E08	2	20	115	14	45	20	−20
PBI_E09	1	17	135	16	45	20	−20
PBI_E10	2	17	135	16	45	20	−20
PBI_E11	1	20	135	16	45	20	−20
PBI_E12	2	20	135	16	45	20	−20
PBI_E13	1	17	115	16	45	20	−20
PBI_E14	2	17	115	16	45	20	−20
PBI_E15	1	20	115	16	45	20	−20
PBI_E16	2	20	115	16	45	20	−20
PBI_E17	1.5	18.5	125	15	45	20	−20

**Table 3:** Input factors (*X_i_*) related to the electrospinning process of PBI solutions.

Parameter	Label	Unit	Low Level (−1)	High Level (+1)	Average Level (0)
Flow rate	*X*_1_	mL/h	1	2	1.5
Ejector voltage	*X*_2_	kV	17	20	18.5
Needle–collector distance	*X*_3_	mm	115	135	125
PBI-solution concentration	*X*_4_	%wt.	14	16	15

**Table 4:** Output responses (*Y_i_*) relating to the microstructural properties of electrospun PBI membranes.

Parameter	Label	Unit
Mean Fibre Diameter (FD)	*Y*_1_	nm
FD Dev. St.	*Y*_2_	nm
Air Permeability	*Y*_3_	cm/s
Porosity	*Y*_4_	%
Beads	*Y*_5_	%

**Table 5:** Average values of microstructural parameters due to increasing polymer solution concentration in PBI.

PBI Solution Concentration [%wt.]	Mean Fiber Diameter [nm]	Air Permeability [cm/s]	Porosity [%]
14	103 ± 25	0.9 ± 0.5	79 ± 5
16	137 ± 30	0.9 ± 0.4	77 ± 8

**Table 6:** Mechanical properties of PBI by casting and electrospinning.

Sample	Young’s Module [GPa]	Tensile Strength [MPa]	Elongation at Break [%]
PBI Cast Film	5.9 ± 1 [36]	120 ± 16 MPa [36]	10 ± 2 [32]
PBI_E12	0.1 ± 0.02	4 ± 0.3 MPa	10 ± 2

**Table 7:** Coded factors (*X*_i_) of the four electrospinning parameters and four output variables (*Y*_i_) (* coded factors).

Sample	*X*_1_ * [-]	*X*_2_ * [-]	*X*_3_ * [-]	*X*_4_ * [-]	*Y*_1_ [nm]	*Y*_2_ [nm]	*Y*_3_ [cm/s]	*Y*_4_ [%]	*Y*_5_ [%]
PBI_E01	−1	−1	1	−1	132	30	1.20	76	1.06
PBI_E02	1	−1	1	−1	125	33	1.52	75	1.07
PBI_E03	−1	1	1	−1	100	25	0.75	83	22.2
PBI_E04	1	1	1	−1	102	25	0.91	82	6.56
PBI_E05	−1	−1	−1	−1	105	28	0.84	82	46.7
PBI_E06	1	−1	−1	−1	89	22	1.11	83	6.79
PBI_E07	−1	1	−1	−1	83	20	0.52	72	52.2
PBI_E08	1	1	−1	−1	89	20	0.68	76	8.23
PBI_E09	−1	−1	1	1	133	33	0.95	74	0
PBI_E10	1	−1	1	1	116	27	1.27	84	49.3
PBI_E11	−1	1	1	1	174	28	0.91	85	0
PBI_E12	1	1	1	1	156	26	0.79	85	0
PBI_E13	−1	−1	−1	1	143	35	1.16	70	14.6
PBI_E14	1	−1	−1	1	132	29	0.89	77	1.89
PBI_E15	−1	1	−1	1	114	28	0.59	76	13.7
PBI_E16	1	1	−1	1	130	40	0.55	68	3.99
PBI_E17	0	0	0	0	139	41	0.59	79	9.81

**Table 8:** Descriptive static models for the three analyzed outputs with the correlation parameters associated with each equation.

Output Variable	Model Equation	*R* ^2^	*R* ^2^ *_adj_*
*Y*_1_	*Y*_1_ *= 120.2 + 9.47 X*_3_ *+ 16.9 X*_4_ *+ 5.01 X*_2_*X*_3_ *+ 7.90 X*_2_*X*_4_ *+ 9.04 X*_2_*X*_3_*X*_4_	0.918	0.8687
*Y*_2_	*Y*_2_ *= 28.11 − 1.440 X*_2_ *+ 2.653 X*_4_ *+ 1.492 X*_1_*X*_2_ *− 0.759 X*_2_*X*_3_ *+ 1.317 X*_2_*X*_4_ *− 2.661 X*_3_*X*_4_ *− 1.442 X*_1_*X*_2_*X*_3_ *+ 1.257 X*_1_*X*_2_*X*_4_ *− 1.472 X*_1_*X*_3_*X*_4_	0.983	0.936
*Y*_3_	*Y*_3_ *= 0.9149 − 0.2031 X*_2_ *+ 0.1231 X*_3_ *− 0.0644 X*_1_*X*_4_	0.833	0.778
*Y*_4_	*Y*_4_ *= 78.11 + 0.831 X*_1_ *+ 2.436 X*_3_ *− 0.692 X*_4_ *− 1.434 X*_1_*X*_2_ *+ 2.957 X*_2_*X*_3_ *+ 0.767 X*_2_*X*_4_ *+ 2.255 X*_3_*X*_4_ *− 1.824 X*_1_*X*_2_*X*_4_ *+ 1.035 X*_1_*X*_3_*X*_4_ *− 0.886 X*_2_*X*_3_*X*_4_	0.978	0.943
*Y*_5_	*Y*_5_ *= 14.01 − 4.54 X*_1_ *− 4.24 X*_3_ *− 3.83 X*_4_ *− 4.13 X*_1_*X*_2_ *+ 8.75 X*_1_*X*_3_ *+ 7.9 X*_1_*X*_4_ *+ 6.13 X*_3_*X*_4_ *− 3.99 X*_1_*X*_2_*X*_3_ *− 4.39 X*_2_*X*_3_*X*_4_	0.954	0.876

## Data Availability

The original contributions presented in this study are included in this article/Supplementary Materials. Further inquiries can be directed to the corresponding authors.

## Supplementary Materials

The following supporting information can be downloaded at: https://www.mdpi.com/article/10.3390/polym17121594/s1, Figure S1. SEM Micrographs of PBI electrospun membrane at 1 mL/h (14 %wt.): samples PBI_E01 and PBI_E05 were obtained at the same flow rate and 17 KV voltage at the ejector; PBI_E03 and PBI_E07 was obtained by varying the voltage (20 kV) and the distance; Figure S2. SEM Micrographs of PBI electrospun membrane at 2 mL/h (14 %wt.): samples PBI_E02 and PBI_E06 were obtained at the same flow rate and 17 kV at the ejector and different needle-collector distance of 135 and 115 mm, respectively; PBI_E04 and PBI_E08 were obtained by varying the voltage at the same 20 kV at the ejector and different needle-collector distance (135–115 mm); Figure S3. SEM Micrographs of PBI electrospun membrane at 1 mL/h (16 %wt.): samples PBI_E09 and PBI_E13 were obtained at the same flow rate and 17 kV at the ejector and a needle-collector distance of 135–115 mm, respectively; PBI_E11 and PBI_E15 were obtained by varying the voltage at the same values of 20 kV and different needle-collector distance (135–115 mm); Figure S4. SEM Micrographs of PBI electrospun membrane at 2 mL/h (16 %wt.): samples PBI_E10 and PBI_E14 were obtained at the same flow rate and 17 kV at the ejector and a needle-collector distance of 135–115 mm, respectively; PBI_E12 and PBI_E16 were obtained by varying the voltage at the same values of 20 kV and different needle-collector distance (135–115 mm); Figure S5. SEM micrograph of PBI electrospun membrane at 15 %wt. working in intermediate conditions (FR = 1.5 mL/h, Vej = 18.5 kV, d = 125 mm, c = 15 %wt.).
