# Supplementary material for: Electrospun Polybenzimidazole Membranes: Fabrication and Fine-Tuning Through Physical and Statistical Approaches
Source: Polymers (Basel). 2025 Jun 6;17(12):1594. doi: 10.3390/polym17121594 (PMC12199289; doi:10.3390/polym17121594)
Supplement: Supplementary file 1 [file polymers-17-01594-s001.zip › polymers-3660678-supplementary.pdf]

SUPPORTING INFORMATIONS

Electrospun Polybenzimidazole Membranes: Fabrication and Fine-tuning through physical and statistical approaches

Emmanuel De Gregorio, Giuseppina Roviello, Valentina Naticchioni, Viviana Cigolotti, Alfonso Pozio, Luis Alexander Hein, Carlo De Luca, Claudio Ferone, Antonio Rinaldi* and Oreste Tarallo *

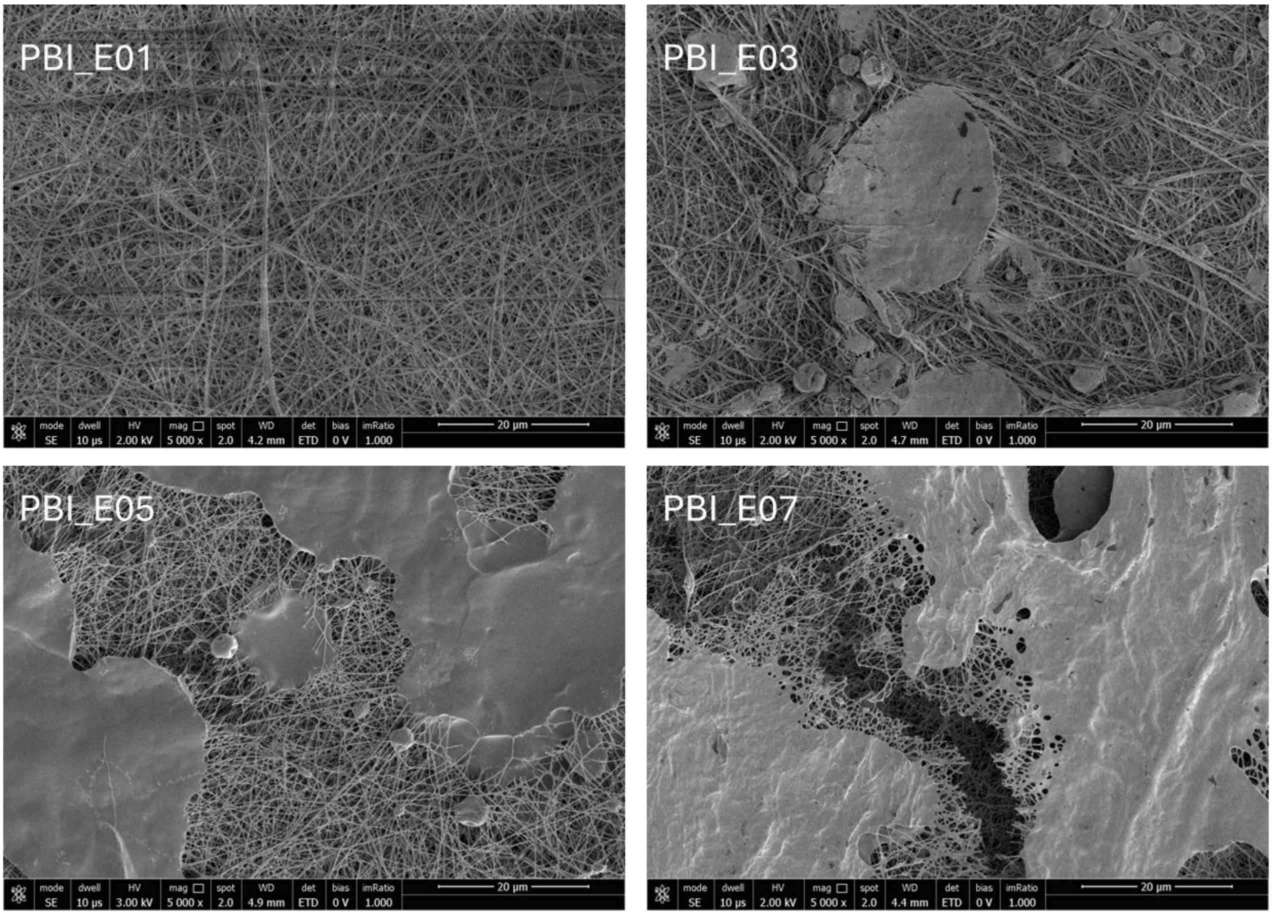

Figure S1. – SEM Micrographs of PBI electrospun membrane at 1 mL/h (14 % wt.): samples PBI_E01 and PBI_E05 were obtained at the same flow rate and 17 KV voltage at the ejector; PBI_E03 and PBI_E07 was obtained by varying the voltage (20 kV) and the distance.

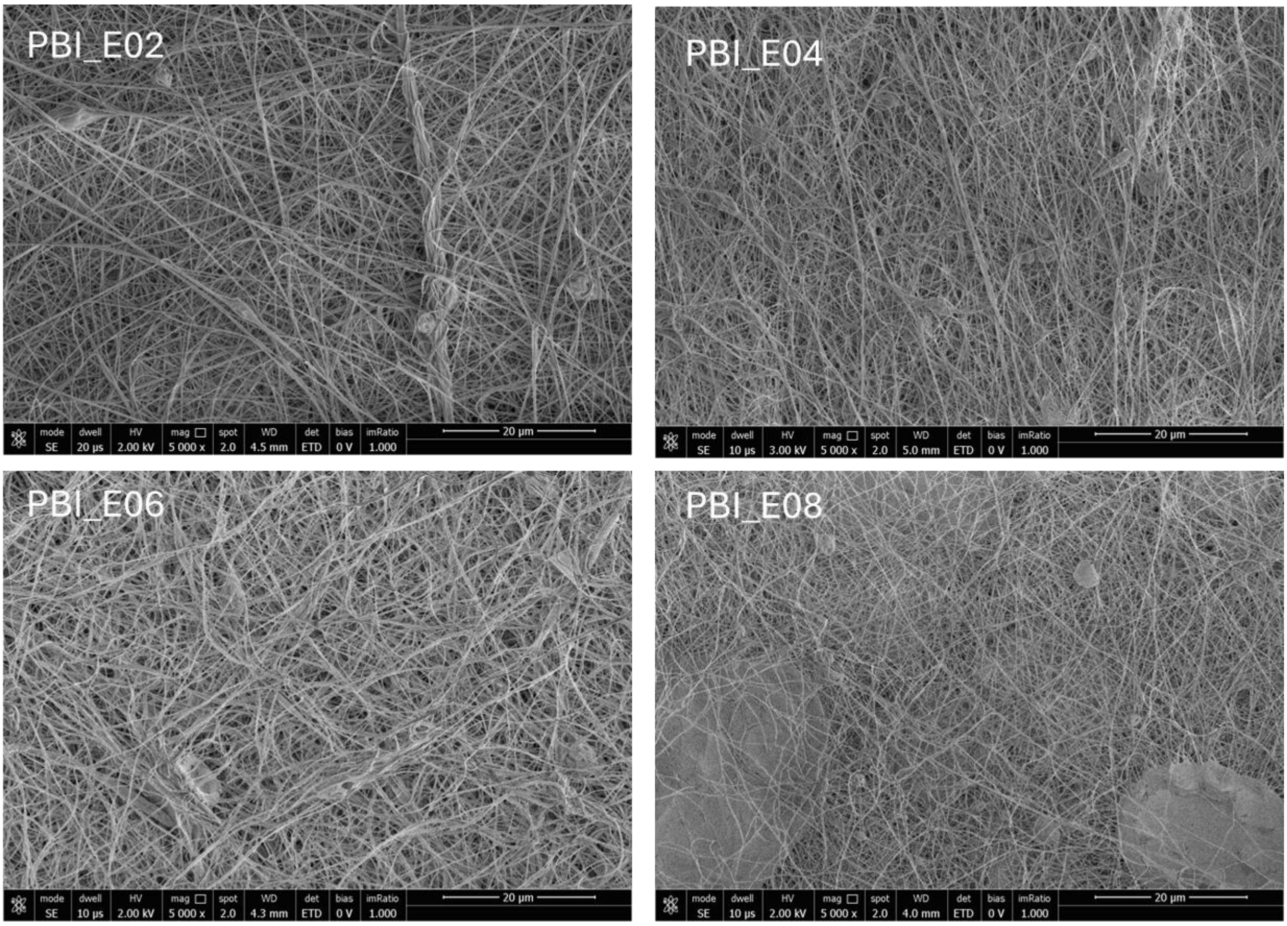

Figure S2. – SEM Micrographs of PBI electrospun membrane at 2 mL/h (14 % wt.): samples PBI_E02 and PBI_E06 were obtained at the same flow rate and 17 kV at the ejector and different needle-collector distance of 135 and 115 mm, respectively; PBI_E04 and PBI_E08 were obtained by varying the voltage at the same 20 kV at the ejector and different needle-collector distance (135-115 mm).

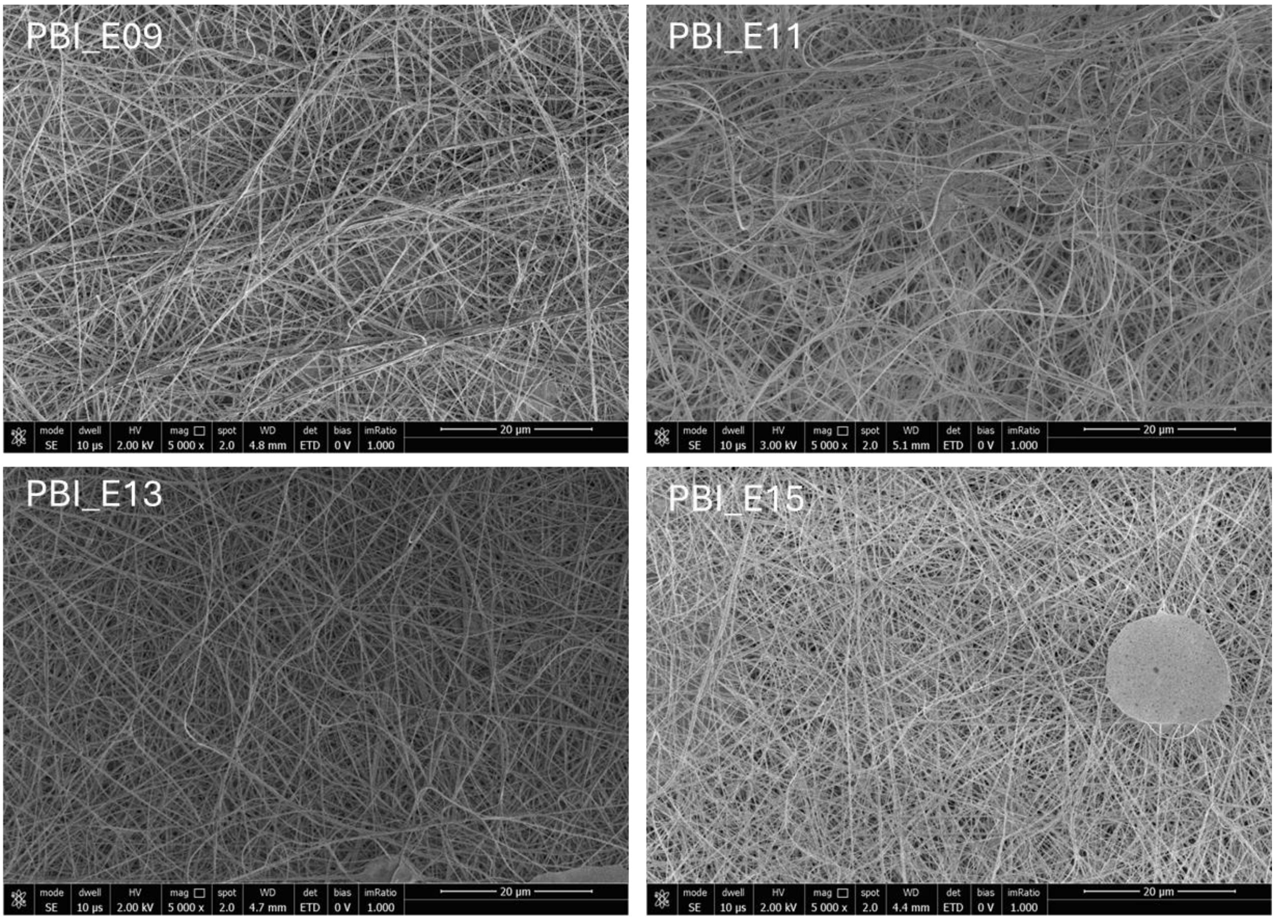

Figure S3. – SEM Micrographs of PBI electrospun membrane at 1 mL/h (16 % wt.): samples PBI_E09 and PBI_E13 were obtained at the same flow rate and 17 kV at the ejector and a needle-collector distance of 135-115 mm, respectively; PBI_E11 and PBI_E15 were obtained by varying the voltage at the same values of 20 kV and different needle-collector distance (135-115 mm).

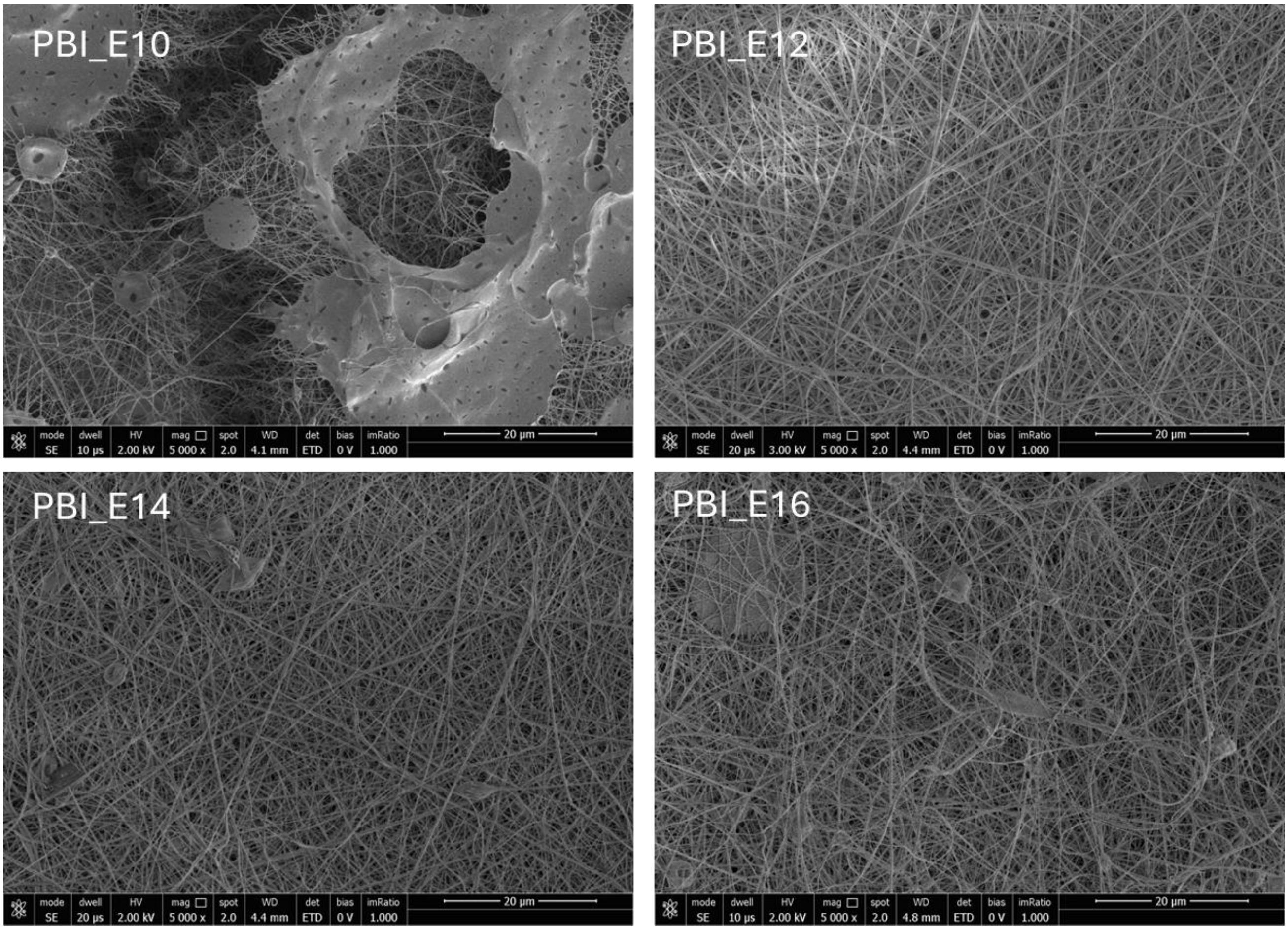

Figure S4. – SEM Micrographs of PBI electrospun membrane at 2 mL/h (16 % wt.): samples PBI_E10 and PBI_E14 were obtained at the same flow rate and 17 kV at the ejector and a needle-collector distance of 135-115 mm, respectively; PBI_E12 and PBI_E16 were obtained by varying the voltage at the same values of 20 kV and different needle-collector distance (135-115 mm).

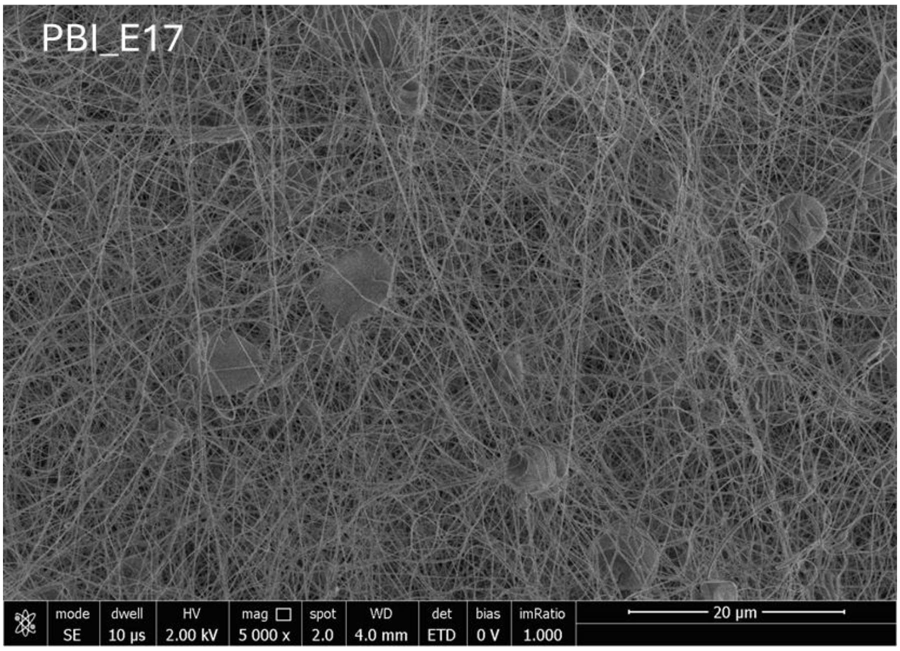

Figure S5. – SEM micrograph of PBI electrospun membrane at 15 % wt. working in intermediate conditions (FR = 1.5 ml/h, V_{ej} =18.5 kV, d = 125 mm, c = 15 % wt.).
